# Photo-Induced Aerobic Oxidation of C–H Bonds

**DOI:** 10.3390/molecules29225277

**Published:** 2024-11-08

**Authors:** Haolin Chen, Feng Liu

**Affiliations:** Department of Chemistry, Fudan University, Shanghai 200438, China

**Keywords:** photocatalysis, aerobic oxidation, C–H bond activation, visible light, green chemistry

## Abstract

The photo-induced aerobic oxidation of C–H bonds has become an increasingly valuable strategy in organic synthesis, offering a green and efficient method for introducing oxygen into organic molecules. The utilization of molecular oxygen as an oxidant, coupled with visible-light photocatalysis, has gained significant attention due to its sustainability, atom economy, and environmentally benign nature. This review highlights the recent advancements in the field, focusing on the development of metal-free and transition-metal-based photocatalytic systems and novel photosensitizers capable of promoting selective C–H bond oxidation. The mechanistic pathways involved in various substrate oxidations, including benzylic, alkyl, alkene, and alkyne C–H bond transformations, are discussed. This review concludes with insights into the potential for integrating photocatalysis with renewable energy sources, positioning photo-induced aerobic oxidation as a cornerstone of sustainable chemical processes.

## 1. Introduction

Oxygen-containing functional groups, such as hydroxyl, carbonyl, carboxyl, sulfonyl, and nitro groups, are widely found in various compounds in nature and confer specific chemical properties to organic compounds [1]. These functional groups allow organic molecules to exhibit different reactivities and selectivities under specific reaction conditions, facilitating further functional group transformations. Oxygen-containing functional groups have various reaction types and participate in many chemical reactions, playing an essential role in organic synthesis. Therefore, studying methods to synthesize these functional groups is of great importance for developing the field of organic synthesis [2].

Oxidation reactions, which introduce oxygen atoms into organic molecules, are the most direct way to construct oxygen-containing functional groups and synthesize oxygenated compounds [3]. These reactions have been widely applied in organic synthesis. However, most traditional methods rely on oxidants such as peroxides and metal oxides, which are often costly and have low atom economy [4]. With the advent of green chemistry, the search for environmentally friendly, non-polluting or low-polluting, efficient, and renewable oxidants that can operate under mild conditions has become increasingly important [5]. Oxygen, a usable oxidant in organic synthesis, stands out for its green, environmentally friendly nature. Additionally, reactions involving oxygen as an oxidant exhibit high atom economy [6]. Oxygen is abundant in nature and easily accessible, and its use in oxidation reactions does not introduce harmful substances, typically producing only water as a byproduct. Using oxygen as an oxidant can reduce environmental pollution and the risk of equipment corrosion [7]. However, the use of oxygen as an oxidant also presents challenges [8]. Some reactions require expensive catalysts and harsh reaction conditions. For example, the oxygen in sulfuric acid production requires vanadium pentoxide as a catalyst [9]. Additionally, in organic reactions, using oxygen as an oxidant can lead to over-oxidation, which prevents the formation of the desired oxidation product [10]. To some extent, these limitations have restricted the widespread application of oxygen as an oxidant [11]. Nevertheless, the potential for oxygen as an oxidant in organic synthesis remains immense, and, by optimizing reaction conditions and methods, it is possible to overcome existing drawbacks and achieve greener, more efficient synthetic processes [12].

Photocatalysis has demonstrated significant advantages in activating oxygen molecules. It is not only environmentally friendly and efficient but also avoids using harmful chemical reagents [13]. Furthermore, photocatalysis can harness sunlight—a renewable energy source—under mild conditions, significantly reducing operational costs. The photocatalytic process is highly tunable, allowing for optimization based on practical needs. It also offers high safety and broad application prospects, making it suitable for environmental protection and new energy, providing a green and sustainable solution for oxygen activation [14]. Below, we briefly overview visible-light photocatalysis in oxidation reactions.

## 2. Visible-Light Photocatalytic Oxidation Reactions

As an inexhaustible energy source in nature, visible light has the advantage of being green and non-polluting. Sunlight, the most common form of green energy, is used by plants in nature to conduct photosynthesis. In 1912, inspired by plant photosynthesis, the organic photochemistry pioneer Ciamician proposed using clean and environmentally friendly photochemical reactions to replace energy-intensive thermal synthesis reactions [15]. If sunlight can be properly utilized as a green energy source, it can significantly alleviate humanity’s energy problems. However, most organic compounds have poor absorption in the visible-light region and cannot directly absorb visible-light energy to undergo reactions. Therefore, we need to use organic or inorganic materials that absorb in the visible-light region as photosensitizers to facilitate reactions, overcoming this barrier and further advancing photochemical research [13].

**Figure 1:**
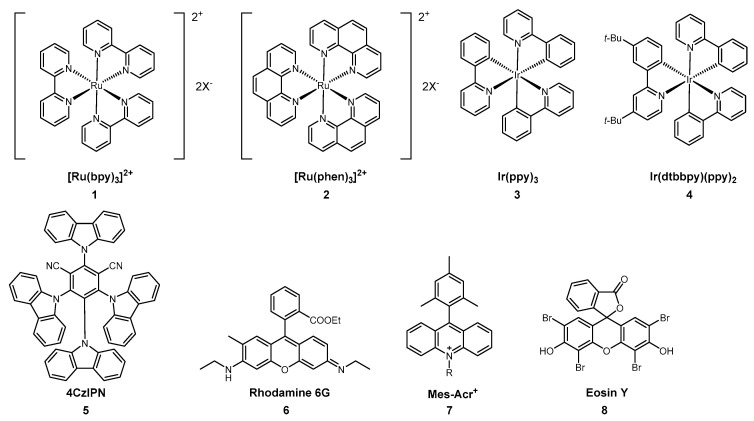
Photosensitizers.

Photosensitizers are molecules or compounds capable of absorbing light of a certain wavelength and reacting under light irradiation (Figure 1) [16]. They play a key role in photochemical reactions. Photosensitizers absorb light within specific wavelength ranges, which vary depending on the specific photosensitizer, typically focusing on the ultraviolet (250–420 nm) and visible-light (400–800 nm) regions. After absorbing light energy, photosensitizer molecules are excited to a higher energy state, where they exhibit high reactivity and can participate in various photochemical reactions. After reacting with a substrate, the photosensitizer typically returns to its ground state, allowing it to catalyze multiple reactions [17].

## 3. Alkane Oxidation

**Scheme 1:**
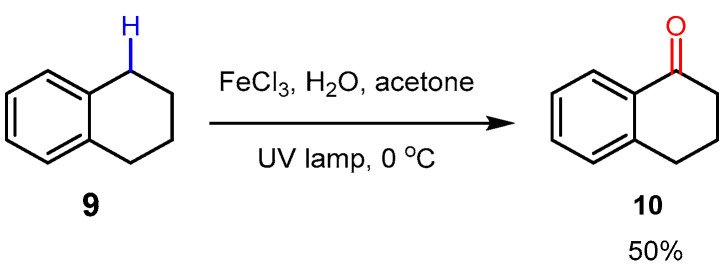
Photo-induced benzylic C–H oxidation.

In 1984, Barbier et al. reported an early example of photocatalytic benzylic C–H bond oxidation, in which secondary C–H bonds in substrate **9** were oxidized to ketone **10** (Scheme 1) [18]. Under UV light irradiation, in the presence of FeCl_3_ and water, tetrahydronaphthalene **9** underwent oxidation to produce 1-tetralone **10**. But, the substrate was limited to indane and diphenylmethane, and the oxidation products of toluene and ethylbenzene yielded less than 10%.

**Scheme 2:**
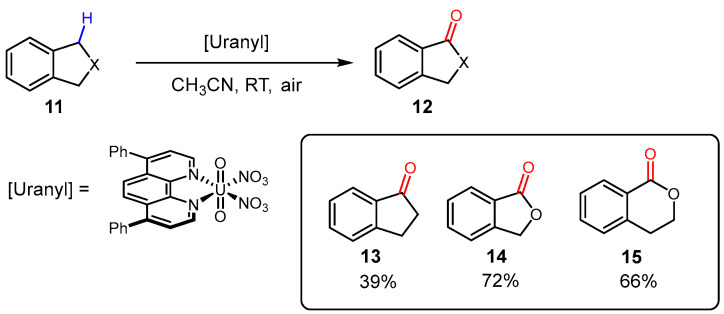
[U^VI^]-catalyzed benzylic C–H oxidation.

In 1996, Bakac reported that an aqueous solution containing toluene, UO_2_^2+^, and oxygen formed benzaldehyde as the major product and a small amount of benzyl alcohol under visible-light irradiation [19]. In 2019, Arnold’s research group used a novel uranyl complex with a phenanthroline ligand to achieve the highly selective catalytic oxidation of benzylic C–H substrates **11** by tuning the electronic structure of the uranyl complex to modulate its photocatalytic performance (Scheme 2) [20].

**Scheme 3:**
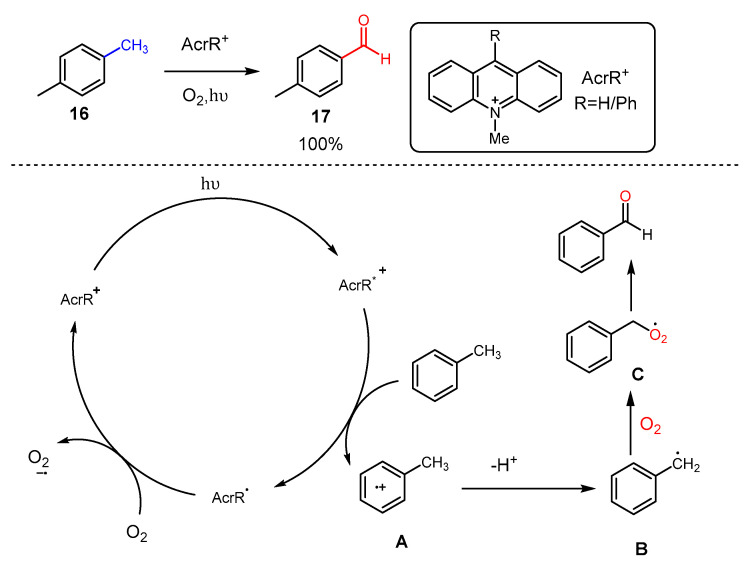
Acridinium-catalyzed oxidation of toluene substrates.

In 2003, Fukuzumi’s group reported the photocatalytic oxidation of substituted toluene using oxygen (Scheme 3) [21]. In this reaction, using a mercury lamp (λ > 300 nm) and tetrafluorodicyanobenzene as the photocatalyst, p-cyanotoluene was oxidized to p-cyanobenzaldehyde by oxygen; using 9-phenyl-10-methylacridinium ion (Acr^+^-Ph) as the photocatalyst, p-xylene **16** was oxidized to p-methylbenzaldehyde **17**.

**Scheme 4:**
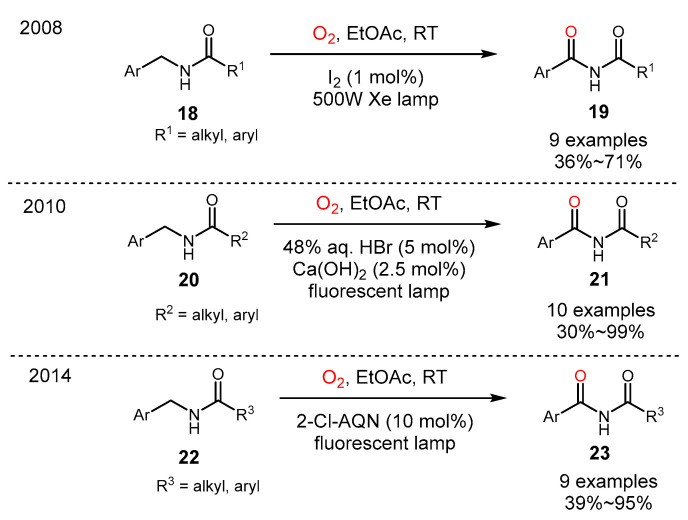
Oxidation of benzylic C–H bonds under different conditions.

In 2008, Itoh’s group reported a photo-promoted benzylic C–H bond oxidation, wherein substrates **18** with various substituents was oxidized at the benzylic position to the corresponding carbonyl compound **19** at the benzylic position with good yields (Scheme 4) [22]. This reaction required only a catalytic amount of iodine to catalyze the oxidation of substrates by oxygen. In 2010, Itoh’s group proposed that hydrobromic acid and calcium hydroxide could also catalyze this oxidative carbonylation reaction. Unlike previous reactions, which required a 500 W xenon lamp as a light source, this reaction proceeded under ordinary incandescent light irradiation [23]. In 2014, they published another study in which 2-chloroanthraquinone catalyzed benzylic C–H bond oxidation, achieving good yields and demonstrating good functional group tolerance across various substituted substrates **22** [24].

**Scheme 5:**
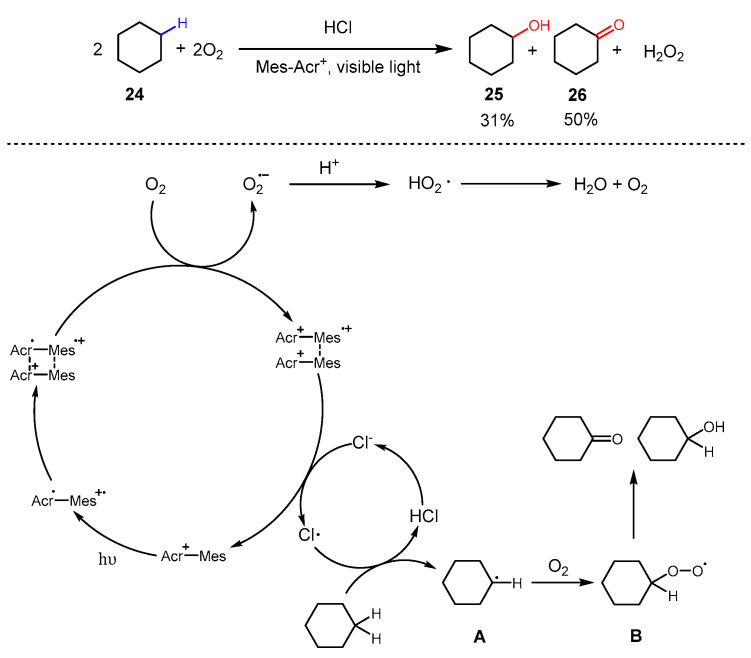
Acridinium-ion-catalyzed oxidation of cycloalkanes.

In 2011, Fukuzumi’s group used acridinium ion as the photocatalyst to achieve cyclohexane **24** oxidation at room temperature (Scheme 5) [25]. Mechanistic studies showed that the reaction involves hydrogen abstraction from cyclohexane by a chlorine radical, generating an alkyl radical. This alkyl radical subsequently reacts with oxygen to form the corresponding alcohol **25** and ketone **26** products.

**Scheme 6:**
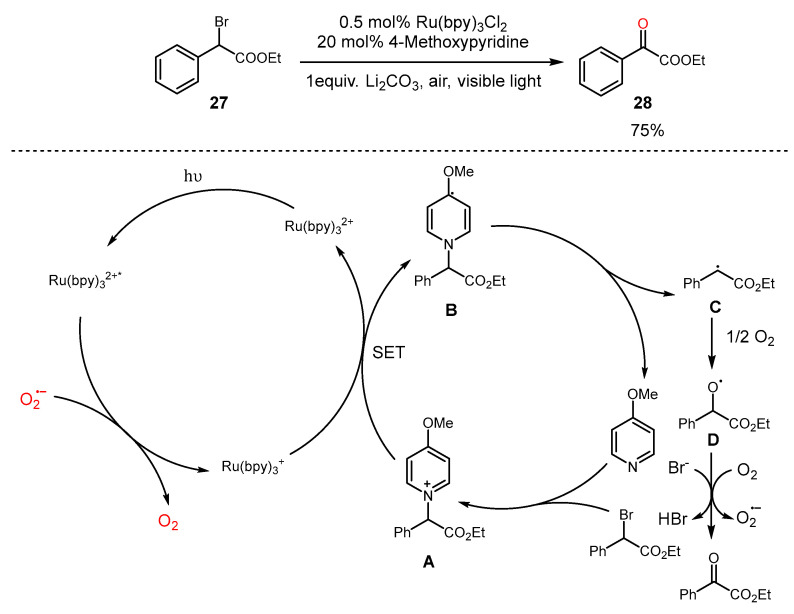
[Rh(II)]-catalyzed oxidation of benzylic halides.

In 2011, Ning Jiao’s group reported a visible-light- and ruthenium-salt-catalyzed oxidation of benzylic halides **27** (Scheme 6) [26]. The proposed mechanism begins with nucleophilic attack by 4-methoxypyridine to form a quaternary ammonium salt, which undergoes the single-electron transfer (SET) oxidation of Ru(I) to generate a radical intermediate. The 4-methoxypyridine then undergoes elimination to regenerate the catalyst while forming an alkyl radical intermediate, which undergoes subsequent oxidation reactions.

**Scheme 7:**
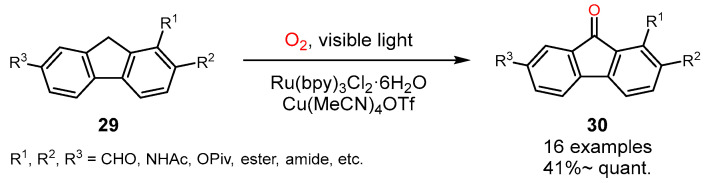
[Ru(II)]-catalyzed oxidation of 9H-fluorene derivatives.

In 2014, Kanai’s group reported a light-promoted ruthenium-salt-catalyzed oxidation reaction. This reaction was compatible with that of various substituted 9*H*-fluorene derivatives **29**, yielding 9-fluorenone **30** products (Scheme 7) [27].

**Scheme 8:**
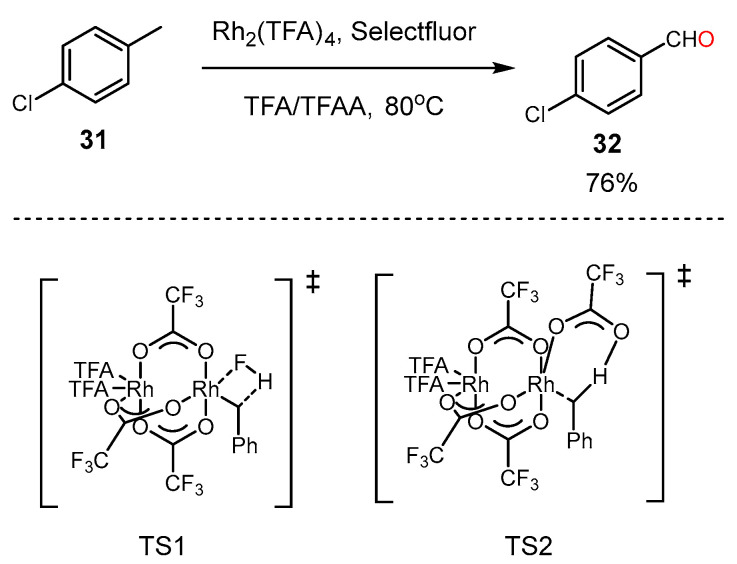
[Rh(II)]-catalyzed oxidation of toluene substrates to aldehydes. ‡ means transition state, which was abbreviated by TS1 and TS2.

In 2015, a joint effort by Lan’s and Rao’s groups resulted in the oxidation of aryl alkanes **31** using rhodium complexes as catalysts (Scheme 8) [28]. Various toluene derivatives **31** were oxidized to the corresponding benzaldehyde products **32** with good yields. Through computational modeling, the researchers proposed two possible mechanisms involving the key intermediate Rh_2_(TFA)_4_. In the first mechanism, Rh_2_(TFA)_4_ activates the C–H bond of the methyl group in the presence of fluoride, forming a four-membered ring transition state (TS1), followed by HF elimination to promote further reaction. In the second mechanism, trifluoroacetic acid induces C–H activation via coordination and hydrogen bonding, forming transition state TS2.

**Scheme 9:**
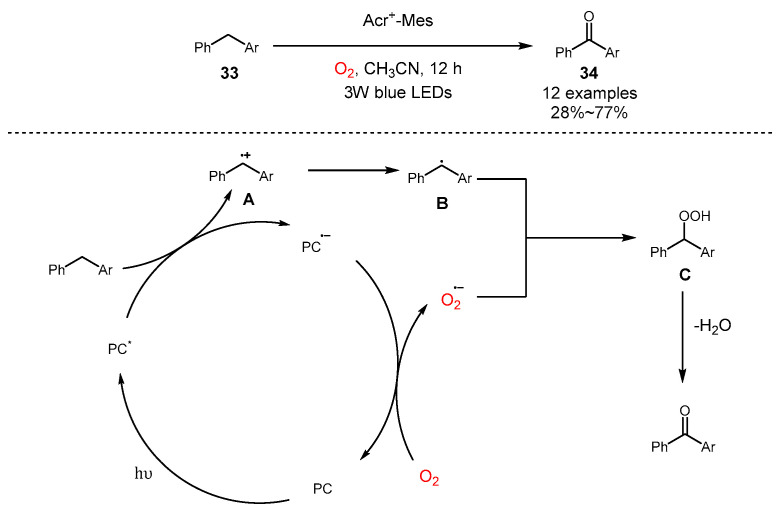
Acridinium-catalyzed sp^3^ benzylic C–H bond oxidation.

In 2015, Lei’s group reported a visible-light-mediated sp^3^ benzylic C–H oxidation reaction (Scheme 9) [29]. The reaction also utilized Acr^+^-Mes as a catalyst, achieving moderate-to-good yields of ketone derivatives **34** from various substrates **33**. Based on previous studies and experimental results, they proposed a mechanism in which the photocatalyst Acr^+^-Mes is excited by blue light to generate the excited state [Acr^+^-Mes]*, which undergoes a SET process with the substrate to form a radical cation and [Acr^+^-Mes]•. [Acr^+^-Mes]• is subsequently oxidized back to its initial state by oxygen, thus completing the photocatalytic cycle. The radical cation loses a proton to form a radical, which then combines with O_2_ or O_2_•^−^ to generate a peroxide intermediate, which is dehydrated to form the ketone.

**Scheme 10:**
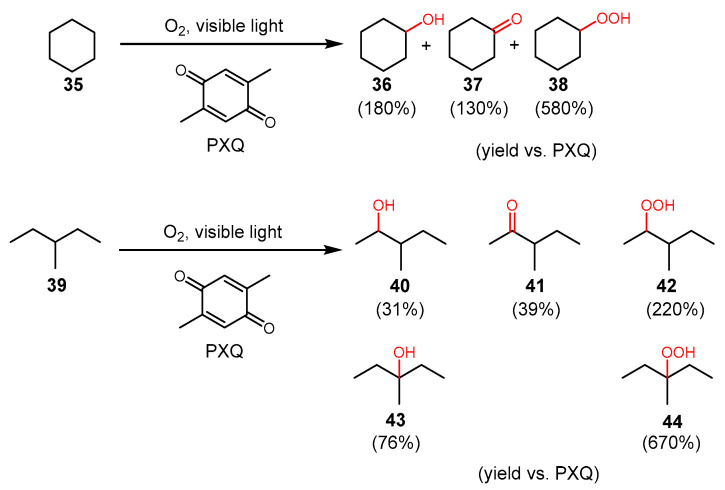
Benzoquinone-catalyzed oxidation of alkanes.

In 2016, Fukuzumi’s group discovered that benzoquinone can also act as a catalyst for the oxidation of cyclohexane **35**, yielding a mixture of peroxides **38** as the major products (Scheme 10) [30]. The reaction of linear alkanes produced more complex mixtures with products from multiple positions.

**Scheme 11:**
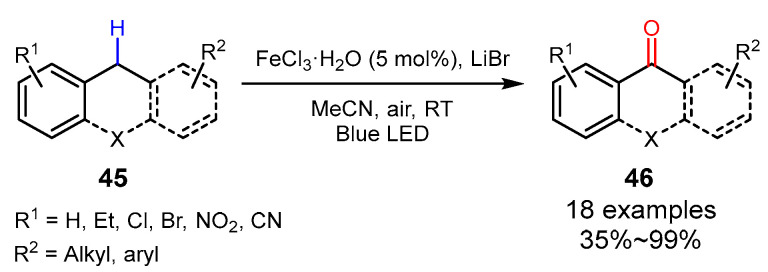
Photocatalytic oxidation of benzylic secondary carbon to ketones.

In 2018, Jiang’s group developed a method for oxidizing secondary benzylic C–H bonds to ketones **46**. Under blue-light irradiation, in the presence of FeCl_3_·6H_2_O and LiBr, substrates **45** such as ethylbenzene, fluorene, and oxanthrene were oxidized at the benzylic position to yield the desired products **46** with moderate-to-high yields (Scheme 11) [31]. Compared to reactions using FeCl_3_ alone, adding an equal amount of LiBr improved the reaction, producing the desired oxygenated products with higher yields. The authors proposed that the mechanism did not involve HAT by Cl· generated from photoexcited FeCl_3_ but instead proceeded via SET from the substrate to photoexcited iron. This was confirmed by the fact that the same oxidation reaction proceeded well in the presence of Fe(NO_3_)_2_ and FeSO_4_. Since FeCl_3_ as a photosensitizer can produce Cl·, both HAT and SET mechanisms are possibly involved in this reaction.

**Scheme 12:**
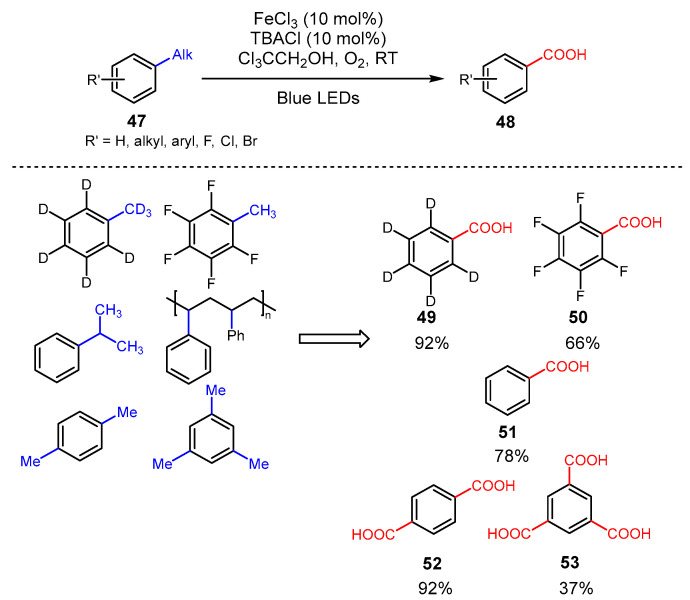
Photocatalytic oxidative cleavage of alkyl aromatics.

In 2021, Zeng’s group reported that inexpensive and readily available ferric chloride could serve as a photocatalyst to mediate the oxidative cleavage of aromatic hydrocarbons, yielding benzoic acid derivatives **48** (Scheme 12) [32]. Using only ferric chloride as a photocatalyst, various substituted toluenes **47** were oxidized under 390 nm LED-light irradiation, producing benzoic acids **48**. Isopropylbenzene, cyclohexylbenzene, and other aromatic hydrocarbons also underwent good C-C bond cleavage in this catalytic system, converting to benzoic acid. The proposed mechanism involves iron’s photogeneration of chlorine or oxygen radicals, which abstracts a hydrogen atom to generate alkyl radicals. These alkyl radicals are then captured by oxygen, forming alcohol or peroxide intermediates, which are further oxidized to the final benzoic acid products.

**Scheme 13:**
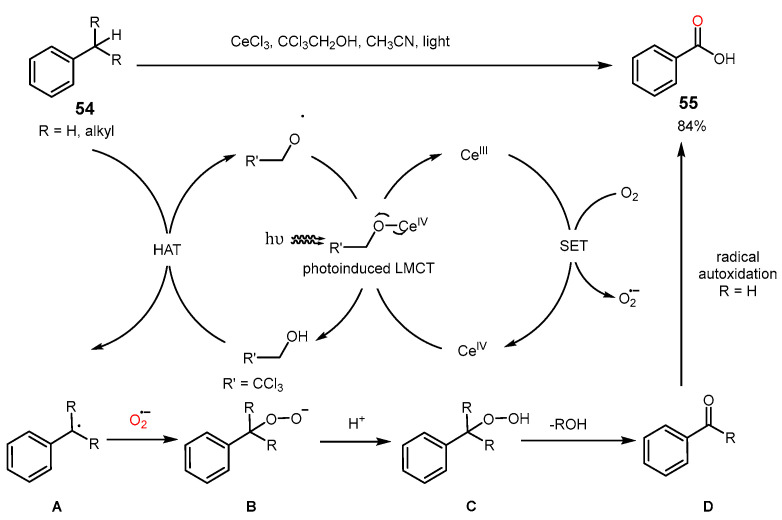
Photocatalytic oxidation of aryl alkanes.

In 2021, Shi’s group reported a method for the oxidation of aryl alkanes (Scheme 13) [33]. Using cerium salts as the photosensitizer, they achieved the oxidation of aryl alkanes **54** under mild conditions, with good to excellent yields across various substrates. They proposed a potential reaction mechanism. In this reaction, cerium salts play two roles: (1) Ce(III) reduces oxygen to form superoxide radical anions via a SET process, and (2) Ce(IV) oxidizes alcohols to alkoxy radicals via a ligand-to-metal charge transfer (LMCT) process. The alkoxy radical then abstracts hydrogen from the substrate, generating the benzyl radical intermediate A. Benzyl radical A reacts with superoxide to form peroxide anion B, which undergoes protonation and subsequent elimination to form the intermediate aldehyde. This aldehyde is further oxidized under light irradiation to form the final carboxylic acid product **55**.

**Scheme 14:**
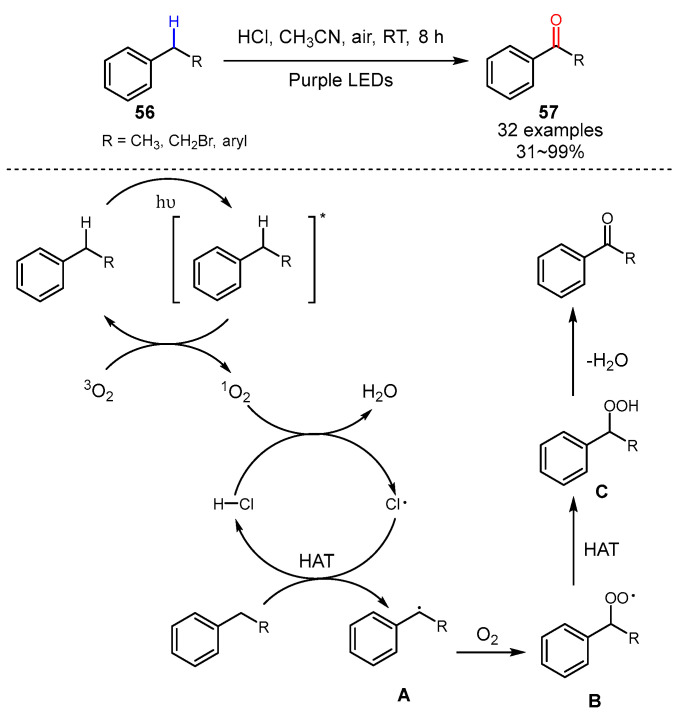
Photocatalytic oxidation of sp^3^ benzylic C–H bonds to ketones.

In 2022, the group led by Qingmin Wang reported a radical-mediated photocatalytic oxidation reaction that could oxidize benzylic positions in alkylbenzenes **56** to carbonyl groups under air and violet-LED-light irradiation (Scheme 14) [34]. Their study revealed that the presence of HCl was necessary for the reaction to proceed. The proposed mechanism suggests that substrate **56** is first excited by light, followed by a reaction between the excited substrate and the triplet oxygen to generate singlet oxygen. The singlet oxygen abstracts a hydrogen atom from HCl, producing a chlorine radical. The chlorine radical then abstracts a hydrogen atom from the substrate to generate a carbon radical, which reacts with oxygen to form the final carbonyl product **57**.

**Scheme 15:**
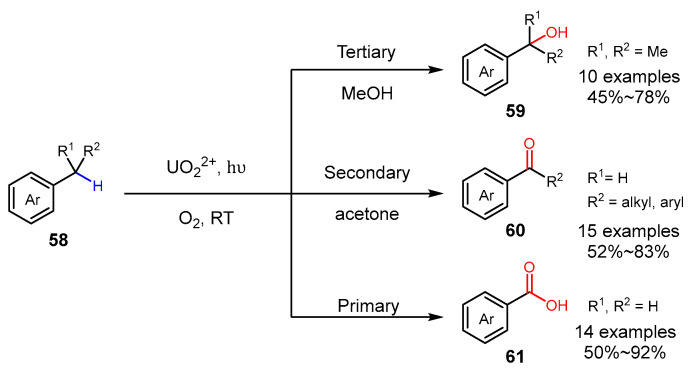
Stepwise oxidation of benzylic positions catalyzed by uranyl cations.

In 2022, Jiang’s group utilized uranyl cations as photosensitizers and achieved the stepwise oxidation of benzylic positions, converting primary, secondary, and tertiary carbons **58** into carboxylic acids **61**, ketones **60**, and aldehydes **59**, respectively (Scheme 15) [35].

## 4. Alkene Oxidation

Alkenes, which are unsaturated hydrocarbons containing C=C double bonds, exhibit unique reactivity due to their functional group structure. In organic synthesis, the double bonds in alkenes can participate in various addition reactions, such as hydrogenation, halogenation, and hydration, as well as oxidation reactions to produce aldehydes, carboxylic acids, and other important compounds. These reactive properties make alkenes key raw materials for the synthesis of polymers, rubber, and fine chemicals. In recent years, the visible-light-catalyzed oxidation of alkenes has developed rapidly, offering efficient catalytic conversion of alkenes into oxygenated compounds and providing new methods and avenues for organic synthesis.

**Scheme 16:**
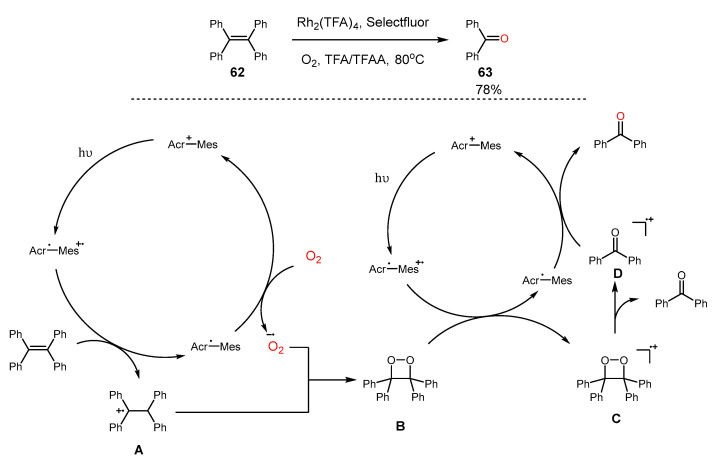
Acridinium-catalyzed oxidation of sp^2^ C–H bonds.

In 2005, Fukuzumi’s group developed a method for the oxidative carbonylation of tetraphenylethylene **62**, leading to C=C bond cleavage and the formation of aldehyde and ketone products **63** (Scheme 16) [36]. In 2006, they found that this reaction was also applicable to diphenylethylene-type compounds [37]. Mechanistic studies ruled out the involvement of singlet oxygen and confirmed via laser flash photolysis and low-temperature EPR experiments that the reaction proceeded via electron transfer from excited [Acr^+^-Mes]* to the alkene. They also isolated 1,2-dioxetane and confirmed its structure using NMR and IR spectroscopy. They proposed that [Acr^+^-Mes] is photoexcited to [Acr^+^-Mes]*, which undergoes SET with the alkene, generating an alkene radical cation. This radical cation couples with O_2_•^−^ to form 1,2-dioxetane, which subsequently undergoes C-O bond cleavage under photocatalysis to yield benzophenone.

**Scheme 17:**
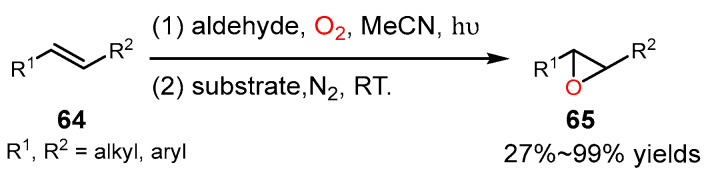
Epoxidation of alkenes.

In 2009, Itoh et al. reported an epoxidation reaction of alkenes using the peroxybenzoic acid generated during the oxidation of benzoic acid (Scheme 17) [38]. This reaction showed moderate-to-excellent yields for various aliphatic and aromatic alkenes **64**, with yields reaching up to 99%.

**Scheme 18:**
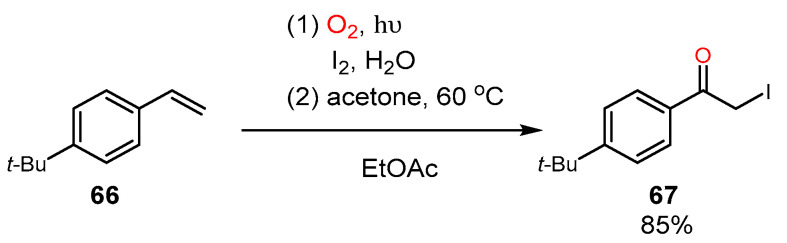
Photocatalytic oxidative carbonylation of styrenes.

In 2010, Itoh’s group reported a photocatalytic carbonylation reaction of styrenes using I_2_ as the catalyst (Scheme 18) [39]. This reaction demonstrated moderate-to-good yields for various styrene derivatives **66** and 1,2-disubstituted alkenes, producing the desired products **67**.

**Scheme 19:**
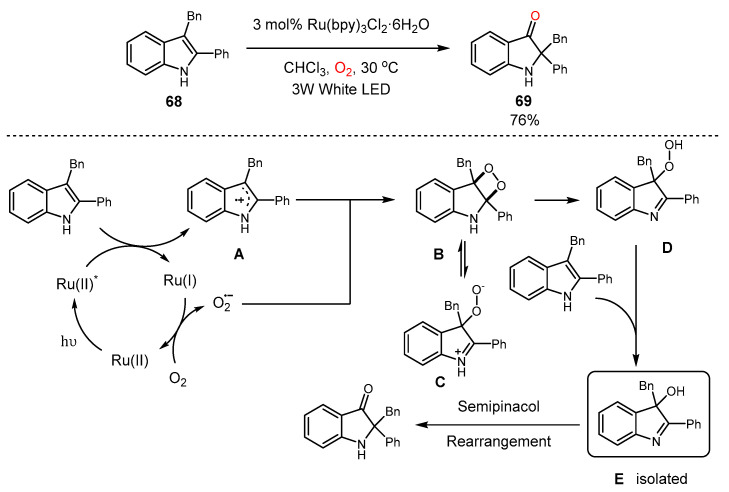
Photocatalytic oxidation–Pinacol rearrangement.

In 2014, Xiao’s group reported a visible-light-induced photocatalytic oxidation–Pinacol rearrangement reaction (Scheme 19) [40], in which indoles **68** were converted into 2,2-disubstituted indole-3-ones **69** under mild conditions using Ru(bpy)_3_Cl_2_ as the catalyst. The indole substrate **68** was first oxidized to form the cation radical **A**, facilitated by photo excited Ru(II)* through a reductive quenching pathway. The resulting Ru(I) species is then reoxidized by molecular oxygen to regenerate Ru(II) and produce a superoxide radical anion. This superoxide radical anion can subsequently react with **A** to yield intermediates **B** or **C**. Proton transfer to form peroxide **D**, O–O bond cleavage to give the tertiary alcohol **E,** then chemospecific semipinacol rearrangement occurred to produce product **69.**

**Scheme 20:**
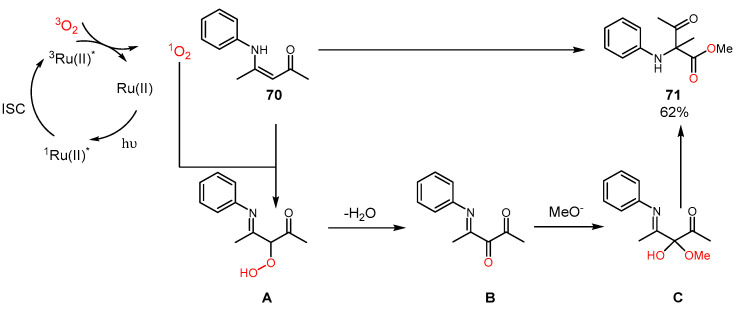
Rearrangement reaction of secondary enaminones.

In the same year, Li’s group reported a similar rearrangement reaction of secondary enaminones (Scheme 20) [41]. In this reaction, the secondary enaminones **70** were oxidized by singlet oxygen, followed by addition to an alcohol and a 1,2-acyl migration. The reaction proceeds through the generation of singlet oxygen from excited ^1^Ru(II)*, which is produced via intersystem crossing (ISC) from the excited ^3^Ru(II)* after light absorption.

**Scheme 21:**
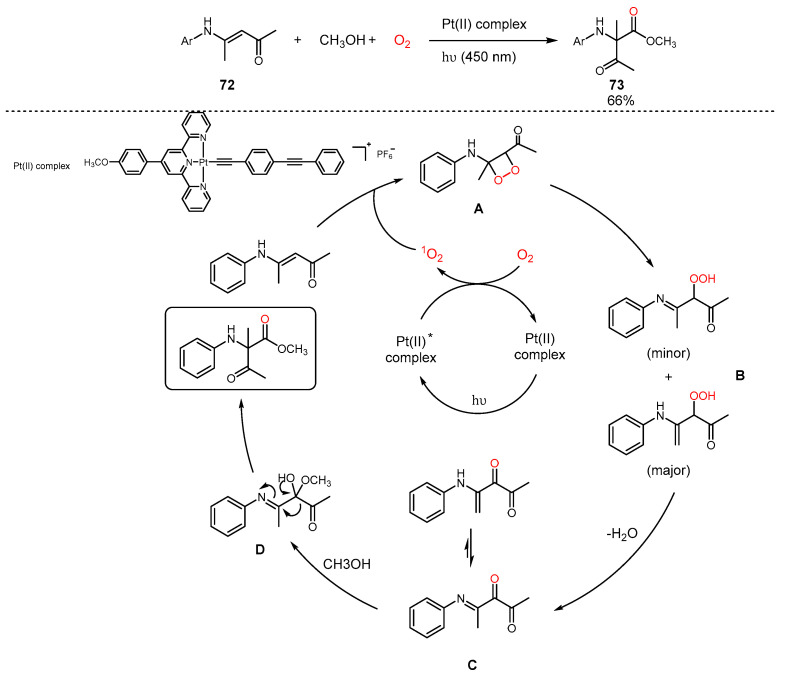
Photocatalytic 1,2-acyl migration reaction.

That same year, Wu’s group reported a similar 1,2-acyl migration reaction (Scheme 21) [42], using a designed platinum complex as the photocatalyst. This reaction was also catalyzed via a singlet oxygen mechanism.

**Scheme 22:**
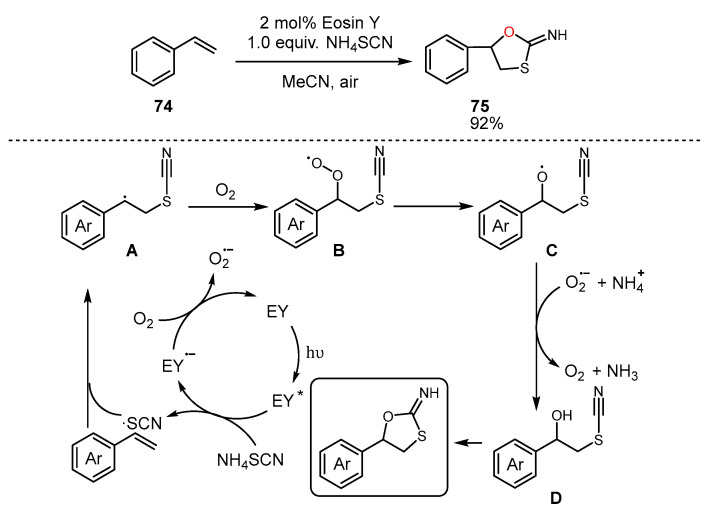
Visible-light-catalyzed difunctionalization of styrene derivatives.

In 2015, Yadav and colleagues reported a visible-light-mediated difunctionalization of styrene **74**, yielding 5-aryl-2-imino-1,3-oxathiolanes **75** in the presence of Eosin Y (EY) (Scheme 22) [43]. Optimization experiments showed that green LEDs were more effective, suggesting that higher-intensity green light increased the photocatalytic activity of EY. The authors proposed a radical addition pathway, wherein the thiol radical adds to the styrene **74**, followed by nucleophilic addition to produce the target product **75**.

**Scheme 23:**
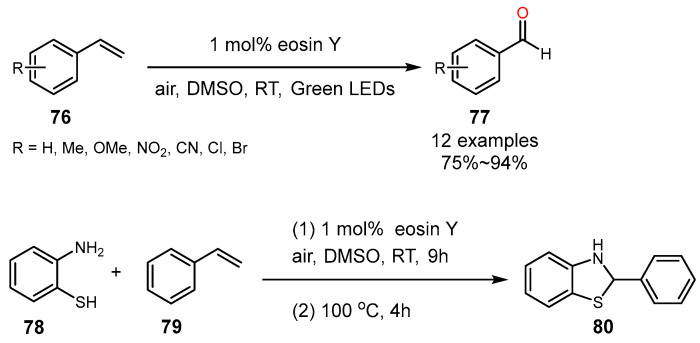
Visible-light-catalyzed oxidative cleavage of styrene derivatives.

That same year, Yadav’s group also reported a reaction in which Eosin Y catalyzed the oxidative cleavage of C=C bonds in styrene derivatives **76** under visible light, with air as the oxidant (Scheme 23) [44]. This reaction enabled the efficient oxidation of styrene derivatives **76** and was used to achieve a one-pot synthesis of benzothiazolines **80**.

**Scheme 24:**
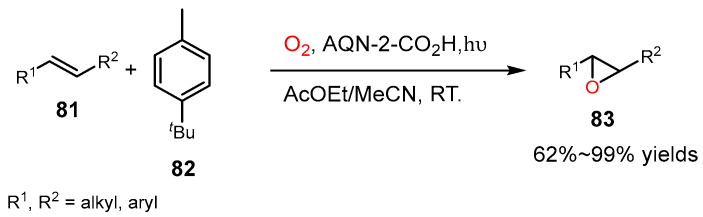
One-pot epoxidation of alkenes.

In 2016, the Itoh group developed a one-pot synthesis method for epoxidizing alkenes **81** (Scheme 24) [45]. In this reaction, toluene **82** was photo-oxidized to form peroxybenzoic acid, which then oxidized alkenes **81** to produce epoxide products **83**.

**Scheme 25:**
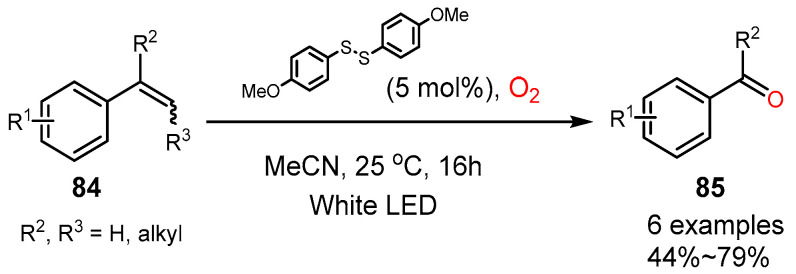
Photocatalytic oxidative carbon–carbon bond cleavage of styrene derivatives.

In 2017, the group led by Wang reported an oxidation reaction of styrene derivatives **84** using substituted disulfides as the photocatalyst, which yielded aldehydes and ketones **85** via carbon–carbon bond cleavage (Scheme 25) [46]. Based on DFT calculations, the authors proposed that the reaction proceeds through the homolytic cleavage of the S-S bond of the activated disulfide under visible light, followed by radical addition of the sulfur radical to the C=C bond.

**Scheme 26:**
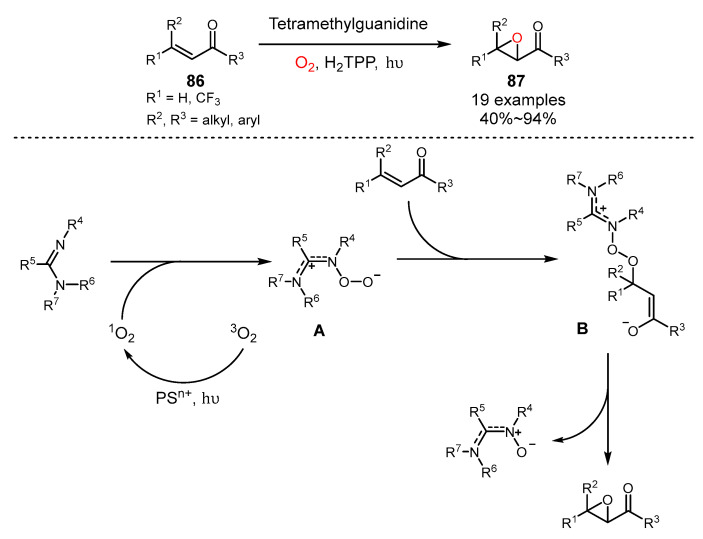
Visible-light-induced epoxidation of α,β-unsaturated ketones.

In 2018, Meng’s group published a visible-light-induced epoxidation reaction of α,β-unsaturated ketones **86** with yields up to 94% (Scheme 26) [47]. Based on mechanistic studies, the authors proposed the following mechanism: Under light irradiation, the photosensitizer catalyzes the generation of singlet oxygen, which oxidizes amidines to form peroxyamidine intermediates. The peroxyamidine intermediates then oxidize the substrate **86** to yield the epoxide product **87**.

## 5. Alkyne Oxidation

**Scheme 27:**
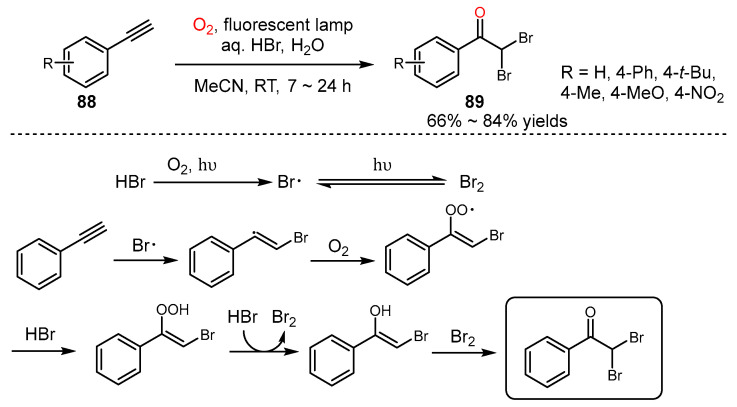
HBr-catalyzed oxidation of terminal alkynes to α,α-dibromo ketones.

In 2010, the Itoh group used HBr as a catalyst to oxidize terminal alkynes **88** to α,α-dibromo ketones **89** (Scheme 27) [48]. This reaction was tolerant to a variety of electron-withdrawing and electron-donating substituents under mild conditions. The proposed mechanism begins with the generation of bromine radicals through the combined action of oxygen and light. These bromine radicals then add to the alkyne substrate **88** to form a vinyl radical intermediate, which reacts with oxygen and is reduced by hydrobromic acid to form an enol. The enol is further brominated to yield the final α,α-dibromo ketone product **89**.

**Scheme 28:**
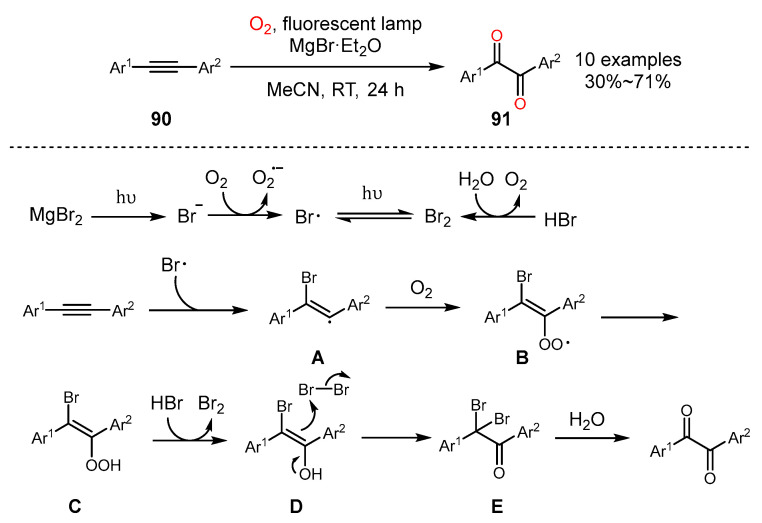
Magnesium bromide–ether complex-catalyzed oxidation of aryl alkynes.

In 2011, Itoh’s group reported an oxidation reaction of aryl alkynes **90** (Scheme 28) [49]. This reaction used a magnesium bromide–ether complex as the photocatalyst. The reaction mechanism was similar to that of the HBr-catalyzed reaction [48], with the main difference being the method of bromine radical generation. The final α,α-dibromo ketone products **E** were hydrolyzed to produce 1,2-dicarbonyl compounds **91**.

**Scheme 29:**
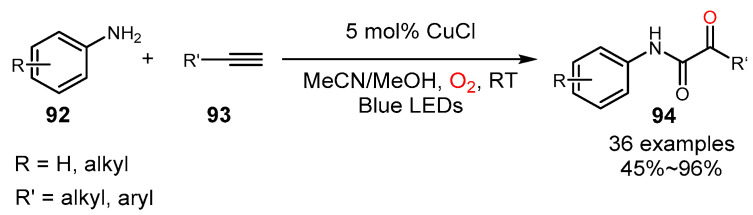
Visible-light-catalyzed one-step synthesis of α-keto amides.

In 2015, Hwang’s group reported a visible-light-mediated, copper(I)-catalyzed C-N coupling reaction between anilines **92** and terminal alkynes **93**, yielding α-keto amides **94** in one step (Scheme 29) [50].

**Scheme 30:**
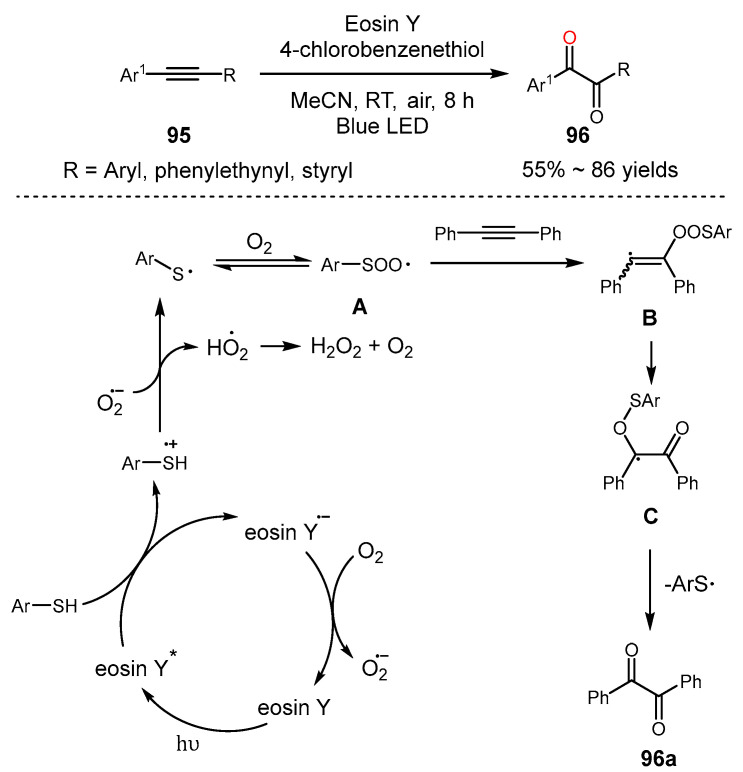
Photocatalytic oxidative dicarbonylation of alkynes.

In 2016, Peipei Sun’s group reported a visible-light-catalyzed alkyne oxidation reaction using Eosin Y as the photosensitizer. This reaction enabled the oxidation of alkynes **95** to 1,2-diones **96** under metal-free conditions, using oxygen as the oxidant (Scheme 30) [51]. The authors proposed the following mechanism: Eosin Y is excited by visible light to eosin Y* and undergoes single-electron oxidation of thiophenol to generate the thiophenol radical cation and Eosin Y radical anion. The Eosin Y radical anion is oxidized by oxygen to produce superoxide anions (O_2_•^−^). The thiophenol radical cation undergoes deprotonation by O_2_•^−^ to form a thiophenol radical. This radical then combines with oxygen, and the resulting intermediate reacts with 1,2-diphenylethyne **95** to generate a vinyl radical. The vinyl radical undergoes rearrangement, eliminating the thiophenol radical and forming the final 1,2-diketone product **96**.

## 6. Conclusions and Perspectives

In conclusion, the photo-induced aerobic oxidation of C–H bonds has emerged as a powerful and green strategy in organic synthesis, providing efficient and selective oxidation methods under mild conditions. The use of oxygen as the terminal oxidant offers several advantages, such as high atom economy and environmentally benign byproducts, primarily water. Through the development of visible-light photocatalysis, significant advances have been made in activating otherwise inert C–H bonds, enabling the formation of valuable oxygen-containing functional groups without the need for expensive or toxic oxidants.

Despite the considerable progress made in this field, several challenges remain. One of the key obstacles is controlling the selectivity of oxidation to prevent over-oxidation, particularly in reactions involving complex molecules or sensitive functional groups. Additionally, although the development of metal-free photocatalysts and transition-metal complexes has broadened the scope of C–H oxidation reactions, the scalability and practicality of these methods for industrial applications need further improvement. Developing more robust and widely applicable photocatalytic systems that can operate efficiently under ambient conditions is essential for their future implementation. In the future, further exploration of reaction mechanisms, including detailed studies on reactive intermediates, will provide deeper insights into the pathways of C–H bond activation and oxidation. Moreover, integrating photocatalytic oxidation systems with renewable energy sources, such as solar energy, holds great promise for sustainable chemical manufacturing. By combining innovative catalyst design with green energy approaches, the photo-induced aerobic oxidation of C–H bonds could become a cornerstone of environmentally friendly and economically viable synthetic chemistry.
